# Factors associated with awareness of breast cancer among women of reproductive age in Lesotho: a national population-based cross-sectional survey

**DOI:** 10.1186/s12889-023-15443-y

**Published:** 2023-03-31

**Authors:** Agani Afaya, Milipaak Japiong, Kennedy Diema Konlan, Solomon Mohammed Salia

**Affiliations:** 1 Mo-Im Kim Nursing Research Institute, College of Nursing, Yonsei University, Seoul, South Korea; 2 Department of Nursing, School of Nursing and Midwifery, University of Health and Allied Sciences, Ho, Ghana; 3 School of Nursing, Faculty of Graduate Studies, York University, York, Canada; 4 Department of Public Health, School of Nursing and Midwifery, University of Health and Allied Sciences, Ho, Ghana; 5 Graduate School of Medical Sciences, Research Institute SHARE, University of Groningen, Groningen, Netherlands

**Keywords:** Breast cancer, Awareness, Reproductive women, Lesotho

## Abstract

**Background:**

Breast cancer is a leading cause of cancer mortality and a major public health problem. The growing number of breast cancer-related deaths has been largely attributed to a lack of awareness of the disease among women. Whilst there have been frequent campaigns promoting breast cancer awareness, evidence suggests that women still lack awareness. Therefore, this study assessed the prevalence and factors associated with the awareness of breast cancer among women of reproductive age in Lesotho.

**Methods:**

We used population-based cross-sectional data from the 2014 Lesotho Demographic and Health survey. A total of 6,620 women of reproductive age were included in the analysis. The outcome variable was awareness of breast cancer. Women who heard about breast cancer were considered to be aware of the disease. Multilevel binary logistic regression models were fitted to determine the factors associated with breast cancer awareness among women.

**Results:**

The level of awareness of breast cancer was 86.8% (95% CI: 85.5, 87.9). Women aged 45–49 years [adjusted odds ratio (AOR) = 2.87, 95% confidence interval (CI): 1.83, 4.48], married women [AOR = 1.51 (95% CI: 1.19, 1.93)], and women with higher educational level [AOR = 12.56, (95% CI: 4.35, 36.28)] were more likely to be aware of breast cancer. Additionally, women who listened to the radio at least once a week [AOR = 1.96, (95% CI: 1.63, 2.37)], those who read newspapers or magazines [AOR = 1.91 (95% CI: 1.48, 2.46)] and women in the wealthiest group [AOR = 2.55, (95% CI: 1.67, 3.89)] had higher odds of breast cancer awareness. However, women who were in rural areas were less likely [AOR = 0.63, (95% CI: 0.47, 0.84)] to be aware of breast cancer than those in urban areas.

**Conclusion:**

The level of awareness of breast cancer among women of reproductive age in Lesotho was extremely low. We recommend that policymakers, clinicians, and public health practitioners should consider the factors identified in this study when designing and developing intervention programs to improve the awareness of breast cancer among women in Lesotho.

## Background

Recent global cancer estimates indicate that breast cancer is the most commonly occurring cancer with high mortality rates. In 2020, approximately 2.3 million women were newly diagnosed with breast cancer, with 685 000 deaths globally [1, 2]. Breast cancer is a leading cause of cancer mortality in sub-Saharan Africa (SSA) and is, therefore, a major public health problem [1]. In SSA, about 129,000 women were diagnosed with breast cancer in 2020 [3]. The 5-year survival rate estimates are near or below 50%, i.e., one in two women diagnosed with the disease will die within 5 years after diagnosis [3]. The burden of breast cancer is envisaged to worsen in Africa in the absence of much-needed interventions [4] due to changes in fertility, lifestyle changes such as late marriage, and shorter periods of breastfeeding among others [4].

Breast cancer deaths in some high-income countries are reducing whereas mortality rates in Low and Middle-Income Income Countries (LMICs) continue to increase[5–7]. This has been attributed to late-stage diagnosis and limited access to comprehensive breast cancer treatment centers [5, 8]. The increased late diagnosis of breast cancer has been largely attributed to the lack of awareness of the disease among the public [9, 10]. An increased level of breast cancer awareness is key for women to present promptly to health facilities if they have breast cancer and a stimulus for improvement in the uptake of breast screening activities [10–14].

Literature indicates that the awareness of breast cancer among women is low in many sub-Saharan African countries [15, 16] raising many concerns. The unique sociocultural dynamics in Africa and belief systems, including low women’s education level, beliefs in witchcraft, fatalism, and spirituality play a key role in influencing women’s awareness of breast cancer [17]. Nonetheless, having a better understanding of the nature and magnitude of breast cancer and identifying the possible reasons for poor breast cancer awareness in SSA is timely [18]. Whilst there have been frequent campaigns promoting breast cancer awareness, evidence suggests that women still lack awareness [19]. Creating awareness of breast cancer among women aims to increase the number of women who present early to healthcare facilities. A concerted effort is therefore needed to determine the factors that either improve or decrease breast cancer awareness among women.

A recent study conducted in Lesotho reported that only 77.6% of women have ever heard of breast cancer [20]. The low awareness of breast cancer among women coupled with the increasing number of breast cancer cases in Lesotho is critical and it is, therefore, worth examining factors influencing the awareness level of women of the disease. To the best of our knowledge, no study has used nationally representative data to examine the awareness of breast cancer among women of reproductive age in Lesotho. Using representative data is essential to target educational resources for women to ensure the appropriate intervention policies are implemented. Therefore, this study determined the prevalence of, and the factors associated with awareness of breast cancer among women of reproductive age in Lesotho. The findings from this study could help direct future strategies around the promotion of breast cancer awareness and contribute to global efforts to reduce mortality and morbidity related to breast cancer. We hypothesized that: (1) women with lower socioeconomic status and education were less likely to be aware of breast cancer, and (2) women’s awareness of breast cancer may vary by geographical location within Lesotho.

## Methods

### Source of data

The data used for the study was derived from the 2014 Lesotho Demographic Health Survey (LDHS). Lesotho is a small, mountainous, and landlocked country, surrounded by South Africa. The country has a population of about 2.2 million people. Lesotho is administratively divided into 10 districts; each district is subdivided into a number of constituencies, and each constituency is further divided into a number of community councils. Literature indicates that about 97% of women between the ages of 15 and 49 are literate compared to the 80% literacy rate of men [21]. In Lesotho, there has been low women representation in economic decision-making policies which ultimately translates to the limited access of women to enhance gender economic planning that advocates for women’s economic empowerment and well-being [21]. The LDHS is a nationally representative survey that gathers information on a wide range of health-related topics that include maternal and child health, maternal and child nutrition, domestic violence, maternal and child mortality, tuberculosis, malaria, and knowledge about the transmission of HIV/AIDS. The LDHS was implemented by the Lesotho Ministry of Health from 22nd September to 7th December 2014. Technical assistance was provided by Inner City Fund (ICF) International through the United States Agency for International Development (USAID)-funded MEASURE DHS program [22]. In the LDHS, a stratified two-stage cluster sampling design was used to sample participants. In the first stage, 400 clusters (of which 118 were in urban areas and 282 in rural areas) were selected based on an updated version of the 2006 Population and Housing Census of Lesotho. Within the clusters were enumeration areas (EAs). In the second stage, households within EAs were selected through a systematic sampling procedure. In each household, only one woman was interviewed. In the interviewed households, 6,818 eligible women were identified for individual interviews out of which 6,621 women completed the survey, yielding a response rate of 97%. In this study after data cleaning and management, 6620 sample of reproductive-age women were included. Further details of the methodology of the survey can be found in the final report of LDHS [22].

### Study variables

#### Outcome variable

The study’s outcome variable was breast cancer awareness among women. The question was stated as “Have you ever heard of a disease called breast cancer?” and the responses were Yes or No. Those who responded as ‘Yes’ were considered to be aware of breast cancer.

#### Independent variables

Based on theoretical significance and the availability of the variables in the dataset, the authors considered both individual and household-level factors in the study. The selection of the variables was considered based on previous studies and their association with breast cancer [23–25].

The individual-level variables included age (15–19, 20–24, 25–29; 30–34, 35–39, 40–44, 45–49); marital status (never married, married, widowed, cohabitating, widowed, divorced); educational status (no education, primary, secondary, higher); religion (Islam, Hindu, Christianity, no religion); health insurance coverage (no or not sure, yes); parity (null, 1–3, 4 and above); pregnancy status (no or not sure, yes); frequency of reading newspaper or magazine (not at all, less than once a week, at least once a week); frequency of listening to radio (not at all, less than once a week, at least once a week); frequency of watching television (not at all, less than once a week, at least once a week).

The household-level factors were the sex of the household head (male or female); place of residence (urban and rural); wealth index (poorest, poorer, middle, richer, richest); distance to health facility (big problem, not a big problem); region **(**Botha-Bothe, Leribe, Berea, Maseru, Mafeteng, Mohale’s hoek, Quthing, Qacha’s-nek, Mokhotlong, Thaba Tseka).

### Statistical analyses

Data analysis was conducted using Stata software version 16.0 (Stata Corporation, College Station, TX, USA). Cross-tabulation was adopted to examine the distribution of breast cancer awareness among reproductive-age women across the explanatory variables. Also, the level of breast cancer awareness was assessed with confidence intervals. The variables significantly associated with breast cancer awareness were determined using bivariate analysis. Significant variables (*p* < 0.05) in the bivariate analysis were included in the multilevel binary logistic regression. Two-level multilevel binary logistic regression models were built to determine the individual and household level factors associated with breast cancer awareness among reproductive-age women in Lesotho. Reproductive-age women were nested within households in the modeling, and households were also nested within clusters. To account for the unexplained variability at the community level, clusters were hypothesized as random effects.

A total of four models were built to determine the predictors of breast cancer awareness. The first model (Model O) was fitted as an empty model without predictors (random intercept). The second model (model I) included individual-level variables against breast cancer awareness among women of reproductive age. Model II was fitted using the household/community level variables against breast cancer awareness among reproductive-age women. The final model (Model III) was fitted by combining all the explanatory variables versus breast cancer awareness. The loglikelihood-ratio test, Akaike’s Information Criterion (AIC), and Bayesian Information Criteria (BIC) were used to evaluate model fitness and model comparison. A multicollinearity diagnostic test was conducted and none of the explanatory variables had a high Variance Inflation Factor (VIF) necessary for exclusion. The sample was weighted (v005/1,000,000), and the survey set command in Stata was used in the analysis to account for the survey’s complex nature. The result of the regression analysis was presented using an adjusted odds ratio (AOR) with 95% confidence intervals (CIs). Statistical significance was set at p < 0.05. We adhered to the guidelines outlined in the STROBE (Strengthening the Reporting of Observational Studies in Epidemiology) statement [21].

### Ethical consideration

Permission was sought from the MEASURE DHS program to use the dataset. We adhered to all ethical standards and guidelines concerning the use of DHS dataset for publication. Ethical information can be found at http://goo.gl/ny8T6X.

## Results

### Level of awareness of breast cancer

A total of 6620 reproductive-age women were included in the study. The level of breast cancer awareness among women of reproductive age was 86.8% (95% CI: 85.5, 87.9).

### Socio-demographic characteristics of respondents

At the individual level, 21.8% of the women were aged 15–19 years. Approximately 53.6% of the women were married while 51.7% had secondary education. The majority of the women (98.0%) were Christians and 98.0% of them were not covered by health insurance. About 63.3% of the women had never read a newspaper or magazine, 60.3% listened to radio, and 29.4% watched the television at least once a week. At the household/community level, 65.1% of the women had a male as their household head, 63.5% of them resided in the urban areas, 26.8% were from the richest wealth index household, 32.4% had no problem with the distance to health facilities (see Table 1).

### Bivariate association between the awareness of breast cancer and explanatory variables

Table 2 outlines the association between breast cancer awareness among women and the explanatory variables. The study found that age, marital status, educational status, health insurance, parity, frequency of reading newspaper or magazine, frequency of listening to radio, frequency of watching television, wealth index, type of place of residence, distance to health facility, and region (*p* < 0.001), were significantly associated with awareness of breast cancer among women.

**Table 1:** Sociodemographic characteristics of reproductive-age women

Variables	Weighted N	Weighted %	No (%)	Yes (%)
**Age (years)**				
15–19	1,440	21.8	25.8	74.2
20–24	1,326	20.0	14.7	85.3
25–29	1,094	16.5	12.4	87.6
30–34	957	14.5	12.1	87.9
35–39	741	11.2	10.2	89.8
40–44	562	8.5	10.8	89.2
45–49	500	7.5	10.0	90.0
**Marital status**				
Never married	2,191	33.0	19.5	80.5
Married	3,547	53.6	13.7	86.3
Cohabitation	64	1.0	15.1	84.9
Widowed	461	7.0	9.6	90.4
Divorced	357	5.4	14.7	85.3
**Educational status**				
No education	68	1.0	29.6	70.4
Primary	2,554	38.6	23.7	76.3
Secondary	3,419	51.7	10.7	89.3
Higher	579	8.7	1.0	99.0
**Religion**				
Islam	19	0.3	0.0	100.0
Hindu	49	0.7	24.6	75.4
Christianity	6,483	98.0	15.3	84.7
No religion	69	1.0	15.5	84.5
**Health insurance coverage**				
No	6,482	98.0	15.6	84.4
Yes	138	2.0	0.9	99.1
**Parity**				
Null	2,079	31.4	20.2	79.8
1–3	3,543	53.5	12.7	87.3
4 and above	998	15.1	14.8	85.2
**Pregnancy status**				
No or unsure	6,336	96.0	16.7	83.3
Yes	284	4.0	15.4	84.6
**Frequency of reading newspaper or magazine**				
Not at all	4,190	63.3	29.7	70.3
Less than once a week	1,368	20.7	13.2	86.3
At least once a week	1,062	16.0	8.8	91.2
**Frequency of listening to radio**				
Not at all	1,565	23.7	19.5	80.5
Less than once a week	1,060	16.0	7.2	92.8
At least once a week	3,995	60.3	7.8	92.2
**Frequency of watching television**				
Not at all	3,670	55.4	21.1	78.9
Less than once a week	1,006	15.2	8.9	91.1
At least once a week	1,944	29.4	6.0	94.0
**Sex of household head**				
Male	4,309	65.1	15.3	84.7
Female	2,311	34.9	15.5	84.5
**Wealth index**				
Poorest	960	14.5	32.6	67.4
poorer	1,034	15.6	22.8	77.2
Middle	1,244	18.8	14.5	85.5
Richer	1,606	24.3	8.2	91.8
Richest	1,776	26.8	4.1	95.9
**Type of place of residence**				
Urban	2,417	36.5	6.1	93.9
Rural	4,203	63.5	20.0	80.0
**Distance to health facility**				
Big problem	1,689	25.5	22.3	77.7
Not a big problem	4,931	74.5	12.8	87.2
**Region**				
Botha-bothe	385	5.8	22.9	77.1
Leribe	1,064	16.1	8.7	91.3
Berea	893	13.5	8.4	91.6
Maseru	1,862	28.1	7.9	92.1
Mafeteng	576	8.7	9.9	90.1
Mohale’s hoek	519	7.8	17.1	82.9
Quthing	315	4.8	22.7	77.3
Qacha’s-nek	204	3.1	21.2	78.9
Mokhotlong	349	5.3	19.5	80.5
Thaba Tseka	453	6.8	25.0	75.0

### Factors associated with the awareness of breast cancer among women

Table 2 outlines the predictors of breast cancer awareness among women in Lesotho after adjustments for all factors included in the final model (III). The study found that women aged 45–49 years were more likely to be aware of breast cancer than those aged 15–19 years [AOR = 2.87, (95% CI: 1.83, 4.48)]. Married women were more likely to be aware of breast cancer compared to those who were never married [AOR = 1.51, (95% CI: 1.19, 1.93)]. Widowed women were also more likely to be aware of breast cancer than those who were never married [AOR = 2.22, (95% CI: 1.45, 3.39)]. Women with higher educational status were more likely to be aware of breast cancer than those who were not educated [AOR = 12.56, (95% CI: 4.35, 36.28)]. Furthermore, women who frequently listened to radio at least once a week [AOR = 1.96, (95% CI: 1.63, 2.37)], and those who read a newspaper or magazine less than once a week [AOR = 1.91, (95% CI: 1.48,2.46)] were more likely to be aware of breast cancer than those who never listened to radio or read newspapers/magazines. Women in the richest category of wealth index [AOR = 2.55, (95% CI: 1.67, 3.89)] were more likely to be aware of breast cancer compared to those in the poorest category. Women who lived in Berea region had higher odds of breast cancer awareness than those resided in the Botha-bothe region [AOR = 2.48, (95% CI: 1.59,3.89)]. Finally, women who resided in rural areas were less likely to be aware of breast cancer than their counterparts in urban areas [AOR = 0.63, (95% CI: 0.47, 0.84)].

**Table 2:** Fixed and random effect analysis of predictors of breast cancer awareness among women of reproductive-age

Variable	COR [95% CI]	Model O	Model I AOR [95% CI]	Model II AOR [95% CI]	Model III AOR [95% CI]
Fixed effect results					
**Age (years)**					
15–19	1.00		1.00		1.00
20–24	2.02***[1.66,2.44]		1.81***[1.40,2.34]		1.74***1.34,2.24]
25–29	2.45***[1.98,3.04]		2.26***[1.66,3.08]		2.09***[1.54,2.85]
30–34	2.52***[2.00,3.17]		2.64***[1.88,3.69]		2.39*** [1.69,3.34]
35–39	3.07***[ 2.35,4.00]		3.45***[2.36,5.06]		2.98***[2.03,4.39]
40–44	2.86***[ 2.15,3.81]		2.94***[1.96,4.42]		2.52***[1.67,3.80]
45–49	3.13***[ 2.28, 4.29]		3.43***[2.20,5.33]		2.87***[1.83,4.48]
**Marital status**					
Never married	1.00		1.00		1.00
Married	1.52***[1.32,1.76]		1.47**[1.15,1.87]		1.51***[1.19,1.93]
Cohabitation	1.36[0.63,2.91]		0.74[0.31,1.79]		0.75[0.31,1.80]
Widowed	2.29***[1.65,3.17]		2.2*** [1.41,3.29]		2.22***[1.45,3.39]
Divorced	1.40***[ 1.02,1.93]		1.21[0.80,1.81]		1.22[0.81,1.83]
**Educational status**					
No education	1.00		1.00		1.00
Primary	1.35[0.83,2.20]		1.56 [0.91,2.69]		1.43 [0.83,2.45]
Secondary	3.52***[2.16,5.74]		3.86*** [2.20,6.78]		3.147***[1.79,5.53]
Higher	43.36***[15.92,118.08]		19.29***[6.70,55.53]		12.56***[4.35,36.28]
**Health insurance coverage**					
No	1.00		1.00		1.00
Yes	19.80**[2.76,142.05]		4.18 [0.55,31.60]		3.36[0.45,25.28]
**Parity**					
Null	1.00		1.00		1.00
1–3	1.74***[1.50,2.02]		1.15 [0.88,1.49]		1.24 [0.95,1.61]
4 and above	1.46***[ 1.196,1.79]		1.06 [0.74,1.52]		1.23[0.86,1.77]
**Frequency of listening to radio**					
Not at all	1.00		1.00		1.00
Less than once a week	2.76***[2.25, 3.38]		1.84***[1.46,2.32]		1.64***[1.30,2.08]
At least once a week	4.35***[3.74, 5.06]		2.35***[1.96,2.82]		1.96***[1.63,2.37]
**Frequency of reading newspaper or magazine**					
Not at all	1.00		1.00		1.00
Less than once a week	3.13***[2.51, 3.91]		2.01***[1.56,2.59]		1.91*** [1.48,2.46]
At least once a week	2.85***[2.22, 3.65]		1.35*[1.01,1.82]		1.24[0.92,1.66]
**Frequency of watching television**					
Not at all	1.00		1.00		1.00
Less than once a week	2.75***[2.16, 3.49]		1.45** [1.10,1.91]		1.14 [0.86,1.51]
At least once a week	4.20***[3.40, 5.19]		1.87*** [1.45,2.42]		1.14 [0.84,1.53]
**Wealth index**					
Poorest	1.00			1.00	1.00
Poorer	1.63***[1.36,1.96]			1.53*** [1.25,1.89]	1.23[0.99,1.54]
Middle	2.84***[ 2.34,3.46]			2.31*** [1.83,2.91]	1.51** [1.17,1.94]
Richer	5.42***[4.34, 6.78]			3.73*** [2.83,4.92]	1.91*** [1.40,2.59]
Richest	11.34***[8.57, 15.01]			6.80*** [4.76,9.72]	2.55***[1.67,3.89]
**Type of place of residence**					
Urban	1.00			1.00	1.00
Rural	0.25***[0.21, 0.31]			0.66** [0.51,0.88]	0.631**[0.47,0.84]
**Distance to a health facility**					
Big problem	1.00			1.00	1.00
Not a big problem	1.95***[ 1.70,2.25]			1.12 [0.95,1.32]	1.13 [0.95,1.35]
**Region**					
Botha-bothe	1.00			1.00	1.00
Leribe	3.13***[2.29,4.29]			2.37***[1.56,3.59]	2.17***[1.40,3.35]
Berea	3.23***[2.35,4.45]			2.47*** [1.61,3.80]	2.48***[1.59,3.89]
Maseru	3.48***[2.56,4.73]			2.19***[1.45,3.32]	2.09***[1.36,3.23]
Mafeteng	2.69***[1.94,3.73]			2.02** [1.31,3.11]	2.18***[1.39,3.43]
Mohale’s hoek	1.44*[1.08, 1.91]			1.38 [0.93,2.06]	1.33 [0.88,2.01]
Quthing	1.01[0.77,1.33]			0.95 [0.64,1.42]	1.02[0.67,1.55]
Qacha’s-nek	1.10[0.83, 1.46]			1.24[0.83,1.85]	1.47[0.97,2.23]
Mokhotlong	1.22[0.93, 1.62]			1.73** [1.16,2.59]	2.11***[1.39,3.20]
Thaba Tseka	0.89[0.68, 1.16]			1.24[0.84,1.83]	1.25[0.83,1.89]
**Random effect model**					
ICC	---	0.2566744	0.1335156	0.079253	0.0821978
Wald chi-square	---	Reference	530.76***	368.61***	626.32***
**Model fitness**					
Log-likelihood	---	-2674.6471	-2347.7897	-2508.9136	-2293.0963
AIC	---	5353.294	4743.579	5051.827	4664.193
BIC	---	5366.89	4906.728	5167.391	4929.309

Exponentiated coefficients; 95% confidence intervals in brackets; COR = crude odds ratio; AOR = adjusted odds ratios; CI Confidence Interval; ^*^p < 0.05, ^**^p < 0.01, ^***^p < 0.001; 1.00 = Reference category; ICC = Intra-Class Correlation; AIC = Akaike’s Information Criterion, BIC = Bayesian Information Criterion

## Discussion

This study assessed the prevalence and factors associated with awareness of breast cancer among women of reproductive age in Lesotho using secondary data from LDHS. The level of awareness of breast cancer among women was 86.8%. From the multilevel fixed effects analysis, we found that women of reproductive age, older women (aged 45–49 years), married women, women with higher educational status, women who used mass media (frequency of reading newspapers or magazines, and frequency of listening to radio), wealthier women and women living in certain regions were more likely to be aware of breast cancer. Women who resided in rural areas were less likely to be aware of breast cancer.

This study found extremely low level of awareness of breast cancer. Even though we found a low level of awareness, our findings was higher than some studies conducted in the West African sub-region: for example the level of awareness of breast cancer among Ghanaian women was 73% [26], and 41.2% in Niger [27]. On the other hand, our finding was lower as compared to a study conducted in Cameroon with 96.3% awareness level [28]. In eastern and southern Africa, a study by Moodley et al. [29] in South Africa and Uganda on mapping awareness of breast and cervical cancer reported that the awareness of breast cancer was 90.8%, while in Ethiopia the awareness level was 53% [30]. The low awareness of breast cancer among women calls for the need to promote the awareness of breast cancer, especially in vulnerable and at-risk populations. This is central to improving screening habits and early detection [26], a panacea to effective treatment.

Consistent with a previous study [31], increasing age of women was associated with the awareness of breast cancer. The focus of most breast cancer education programs has largely focused on populations that has a high chance of developing breast cancer even though recent reports indicate that younger women should be screened as well [32]. Whilst breast cancer screening is more common among women in the premenopausal and menopausal ages, it is important that given the increasing incidence of the disease among younger population, health education and promotion of breast cancer awareness should target this group as well [32]. Breast cancer education especially in LMICs where healthcare infrastructure is not well developed for treatment of advanced stage of the disease must be done early among women in their reproductive years to promote early detection and treatment [32, 33].

This study revealed that married women were more likely to be aware of breast cancer than those not married. Our finding is consistent with previous research findings [34, 35] where married women had better breast cancer awareness relative to those not married. We may attribute this finding to the fact that married women might have received encouragement and motivation from their partners to seek health care and therefore might have been exposed to breast cancer education. Also, our finding could be attributed to the role of childbirth and its potential to increase access to health information during antenatal and immunization. Our study also revealed that widowed women were more likely to be awareness of breast cancer. It is possible that ever having a partner might have been the reason for the present study finding. These findings suggest that intervention programs targeted at increasing the awareness of breast cancer should pay more intention to unmarried women in order to improve their awareness level especially as they may be at increased risk because of different fertility and breastfeeding patterns.

Higher educational attainment has been known all over the world to be significantly related to women’s awareness of breast cancer [33, 36–38]. In all these studies, it was shown that women’s educational status increased their awareness and the chance of using screening services [13, 33, 36–38]. Women of higher educational status can read and comprehend health-related information compared to the non-educated women. As women’s educational level increases, it correlates with improved socio-economic status, thus eliminating key barriers to the acquisition of health information and healthcare services. This is particularly important because our findings further concur with several other studies [23, 39] that revealed that women of a higher socio-economic status were more likely to have higher breast cancer awareness compared to those of a low socioeconomic status. To curb the breast cancer situation in developing countries, institutions must put measures that target women with lower formal educational and socioeconomic status. Healthcare information about breast cancer must be translated into local languages for easy comprehension by women without formal education.

Our study finding is consistent with studies conducted in Ethiopia [40] and Uganda [41] that revealed that those who had/listened to television or radio were more likely to be aware of breast cancer. This study emphasizes the essence of media information in improving awareness of breast cancer. It is important that diverse and multiple channels are used to disseminate information about breast cancer. These multiple channels should be complementary as both print and electronic media appear to be useful in health education. Consistent with studies conducted in SSA [16] and Turkey [42] women in rural settlements were less likely to be aware of breast cancer therefore health promotion efforts on breast cancer tailored towards rural communities are key in creating awareness about the disease.

### Limitations

There were some methodological limitations. The cross-sectional design employed by DHS makes it impractical to establish causal relationships. Also, breast cancer awareness level was self-reported in this study, hence social desirability and recall bias may have affected the reporting of awareness. Nonetheless, nationally representative data were used in this study making the findings generalizable to women of reproductive age in Lesotho together with the robust statistical analysis.

### Implication for practice and policy

This study provides key determinants of breast cancer awareness among women of reproductive age and these findings have a significant implication: younger women and those with less formal education lack breast cancer awareness. Increasing breast cancer awareness, especially, in vulnerable and at-risk populations is central to improving screening habits and early detection. It is also important for policymakers to drive health promotion interventions targeting rural communities in order to raise their awareness levels. These findings are important for clinicians and policymakers who are involved in the development and implementation of strategies to promote breast cancer awareness. Policymakers addressing health system challenges must implement measures that will promote the elimination of structural barriers to breast cancer-related information. This will involve intensifying media education and information dissemination. The use of divergent and multiple information dissemination sources should remain a focus towards improving awareness related to breast cancer, especially in most deprived areas (rural areas). Also, promoting the discussion of breast cancer issues in primary healthcare facilities will be key in mitigating the deterrent effect of these barriers [43]. Also, incorporating breast cancer education in primary healthcare systems will remain essential in promoting access to this information as distance to health facility was found to influence women’s level of awareness.

## Conclusion

The level of awareness of breast cancer was 86.8% among women of reproductive age in Lesotho which was extremely low. The predictors of women’s awareness of breast cancer included older reproductive age, being in the richest wealth index category, exposure to media (i.e., radio and print media), higher formal education, and being married. It is important that diverse and multiple media channels are used to disseminate information about breast cancer. We recommend that policymakers, clinicians, and public health practitioners should consider these factors identified in this study when designing and developing intervention programs to improve the awareness of breast cancer among women in Lesotho.

## Data Availability

The dataset used for this study is openly available and can be accessed via https://dhsprogram.com/data/.

## Abbreviations

LDHS: Lesotho Demographic Health Survey
SSA: sub-Saharan Africa
LMICs: low- and middle-income countries
USAID: United States Agency for International Development
UNFPA: United Nations Population Fund
PEPFAR: the U.S. President’s Emergency Plan for AIDS Relief
UNICEF: United Nations Children’s Fund, World Bank,
WHO: World Health Organization
HIV: Human Immunodeficiency Virus
AIDS: Acquired Immunodeficiency Syndrome
VIF: Variance Inflation Factor
AIC: Akaike’s Information Criterion
BIC: Bayesian Information Criteria
COR: crude odd ratio
AOR: adjusted odds ratios
CI: Confidence Interval
